# Crystal structure of 14-methyl-11-(3-methyl­phen­yl)-12-oxa-8,14-di­aza­tetra­cyclo­[8.3.3.0^1,10^.0^2,7^]hexa­deca-2(7),3,5-triene-9,13-dione

**DOI:** 10.1107/S2056989015008129

**Published:** 2015-05-07

**Authors:** M. P. Savithri, M. Suresh, R. Raghunathan, R. Raja, A. SubbiahPandi

**Affiliations:** a Department of Physics, Queen Mary’s College (Autonomous), Chennai 600 004, India; b Department of Organic Chemistry, University of Madras, Guindy Campus, Chennai 600 025, India; c Department of Physics, Presidency College (Autonomous), Chennai 600 005, India

**Keywords:** crystal structure, tetra­cyclo, hexa­deca­trienedione, hydrogen bonding

## Abstract

In the title compound, C_21_H_20_N_2_O_3_, the lactone ring adopts an envelope conformation with the quaternary C atom bonded to two other C atoms as the flap. The fused pyrrolidine ring adopts a twisted conformation about the C_q_—N (q = quaternary) bond. In the crystal, inversion dimers linked by pairs of N—H⋯O hydrogen bonds generate *R*_2_^2^(8) loops. The dimers are linked into [110] chains by pairs of C—H⋯O inter­actions, which generate *R*_2_^2^(14) loops.

## Related literature   

For related structures, see: Ramesh *et al.* (2008 ▸); Zhao & Teng (2008 ▸); Bai *et al.* (2009 ▸); Du *et al.* (2010 ▸); Wang *et al.* (2010 ▸). For puckering parameters, see: Cremer & Pople (1975 ▸). 
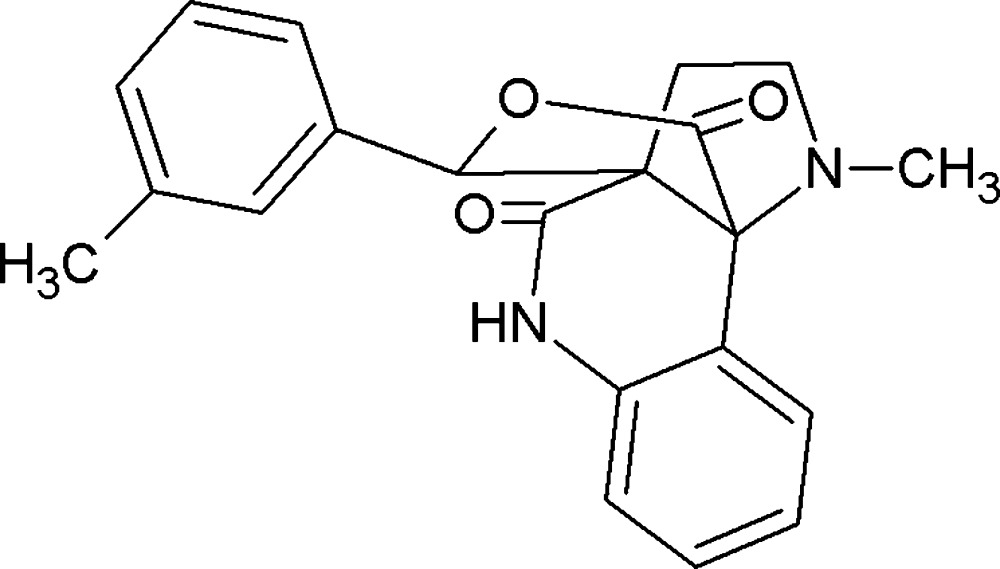


## Experimental   

### Crystal data  

C_21_H_20_N_2_O_3_
*M* *_r_* = 348.39
Monoclinic, 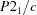

*a* = 10.4772 (7) Å
*b* = 8.6834 (6) Å
*c* = 19.1123 (13) Å
β = 92.490 (2)°
*V* = 1737.2 (2) Å^3^
*Z* = 4
Mo *K*α radiation
μ = 0.09 mm^−1^
*T* = 293 K
0.35 × 0.30 × 0.30 mm

### Data collection  

Bruker Kappa APEXII CCD diffractometer
Absorption correction: multi-scan (*SADABS*; Bruker, 2004 ▸) *T*_min_ = 0.969, *T*_max_ = 0.974
25650 measured reflections
3787 independent reflections
2875 reflections with *I* > 2σ(*I*)
*R*_int_ = 0.024

### Refinement  

*R*[*F* ^2^ > 2σ(*F* ^2^)] = 0.045
*wR*(*F* ^2^) = 0.137
*S* = 1.09
3787 reflections
239 parameters
H atoms treated by a mixture of independent and constrained refinement
Δρ_max_ = 0.29 e Å^−3^
Δρ_min_ = −0.21 e Å^−3^

### 

Data collection: *APEX2* (Bruker, 2004 ▸); cell refinement: *SAINT* (Bruker, 2004 ▸); data reduction: *SAINT*; program(s) used to solve structure: *SHELXS97* (Sheldrick, 2008 ▸); program(s) used to refine structure: *SHELXL97* (Sheldrick, 2008 ▸); molecular graphics: *ORTEP-3 for Windows* (Farrugia, 2012 ▸); software used to prepare material for publication: *SHELXL97* and *PLATON* (Spek, 2009 ▸).

## Supplementary Material

Crystal structure: contains datablock(s) global, I. DOI: 10.1107/S2056989015008129/hb7360sup1.cif

Structure factors: contains datablock(s) I. DOI: 10.1107/S2056989015008129/hb7360Isup2.hkl

Supporting information file. DOI: 10.1107/S2056989015008129/hb7360Isup3.cml

**Fig. 1.**
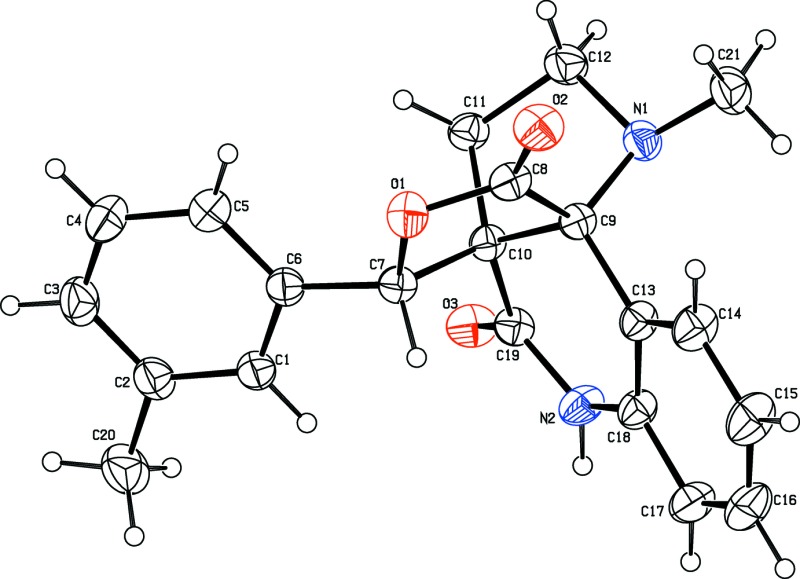
The molecular structure of the title compound with displacement ellipsoids drawn at the 30% probability level.

**Fig. 2.**
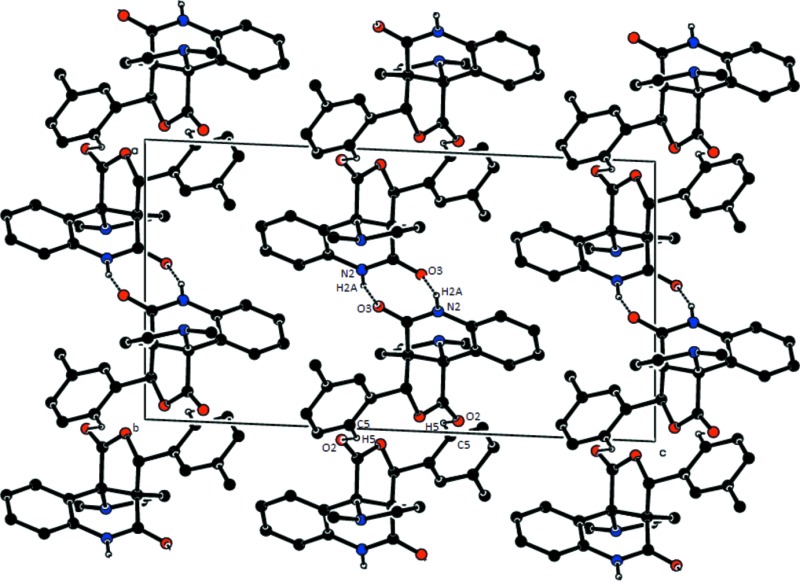
The molecular packing is viewed along the b axis. Dashed lines shows the intermolecular C-H···O and N-H···O hydrogen bonds. H atoms not involved in hydrogen bonding have been omitted for clarity.

. DOI: 10.1107/S2056989015008129/hb7360fig1.tif
The mol­ecular structure of the title compound with displacement ellipsoids drawn at the 30% probability level.

b . DOI: 10.1107/S2056989015008129/hb7360fig2.tif
The mol­ecular packing is viewed along the *b* axis. Dashed lines shows the inter­molecular C-H⋯O and N-H⋯O hydrogen bonds. H atoms not involved in hydrogen bonding have been omitted for clarity.

CCDC reference: 1061495

Additional supporting information:  crystallographic information; 3D view; checkCIF report

## Figures and Tables

**Table 1:** Hydrogen-bond geometry (, )

*D*H*A*	*D*H	H*A*	*D* *A*	*D*H*A*
N2H2*A*O3^i^	0.91(2)	1.93(2)	2.802(2)	159.6(19)
C5H5O2^ii^	0.93	2.55	3.303(3)	138

Symmetry codes: (i) 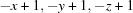; (ii) 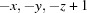.

## supplementary crystallographic information

### S1. Comment

The geometric parameters of the title molecule (Fig. 1) agree well with reported similar structures (Ramesh *et al.*, 2008; Zhao & Teng, 2008; Bai *et al.*, 2009; Du *et al.*, 2010; Wang *et al*., 2010). The five-membered furan ring (atoms O1/C7–C10) adopts an envelope conformation, with atom C10 as the flap, with puckering parameters (Cremer & Pople, 1975), q_2_ = 0.4062 (2) Å and φ_2_ = 246.5 (2)° and the pyrrolidine ring (atoms N1/C9–C12) exhibits a twisted conformation as indicated by the puckering parameters q_2_ = 0.3981 (2) Å and φ_2_ = 22.4 (3)°. The quinoline ring system is roughly planar, with a maximum deviation of 0.315 (2) Å for atom C10 and the plane of the furan ring is oriented at dihedral angles of 72.5 (8) and 77.9 (1)° with respect to the quinoline and pyrrolidine rings, respectively.

In the crystal, hydrogen-bonded chains running along [110] are generated by connecting neighbouring molecules *via* C—H···O and N—H···O hydrogen bonds, forming a chain. Intermolecular C5—H5···O2 hydrohen bonding forms an *R*_2_^2^(14) graph-set dimer (Fig. 2 and Table 1). In addition to this, another graph-set dimer of *R*_2_^2^(8) forms in the unit cell involving N2—H2A···O3 hydrogen bonds (Fig. 2).

### S2. Experimental

A mixture of methyl 2-(hydroxy(m-tolyl)methyl)acrylate (1 mmol), isatin (1.1 mmol) and sarcosine (1.1 mmol) was placed in a round bottom flask and melted at 180°C until completion of the reaction was evidenced by TLC analysis. After completion of the reaction, the crude product was washed with 5ml of ethylacetate and hexane mixture (1:4 ratio) which successfully provided the pure product as colorless solid. The product was dissolved in ethyl acetate and heated for two minutes. The resulting solution was subjected to crystallization by slow evaporation of the solvent for 48 hours resulting in the formation of colourless blocks.

### S3. Refinement

All H atoms were fixed geometrically and allowed to ride on their parent C atoms, with C-H distances fixed in the range 0.93-0.98 Å with *U*_iso_(H) = 1.5*U*_eq_(C) for methyl H 1.2*U*_eq_(C) for other H atoms.

### Crystal data

**Table:**

C_21_H_20_N_2_O_3_	*F*(000) = 736
*M**_r_* = 348.39	*D*_x_ = 1.332 Mg m^−^^3^
Monoclinic, *P*2_1_/*c*	Mo *K*α radiation, λ = 0.71073 Å
Hall symbol: -P 2ybc	Cell parameters from 3787 reflections
*a* = 10.4772 (7) Å	θ = 2.1–27.0°
*b* = 8.6834 (6) Å	µ = 0.09 mm^−^^1^
*c* = 19.1123 (13) Å	*T* = 293 K
β = 92.490 (2)°	Block, colourless
*V* = 1737.2 (2) Å^3^	0.35 × 0.30 × 0.30 mm
*Z* = 4	

### Data collection

**Table:**

Bruker Kappa APEXII CCD diffractometer	3787 independent reflections
Radiation source: fine-focus sealed tube	2875 reflections with *I* > 2σ(*I*)
Graphite monochromator	*R*_int_ = 0.024
ω and φ scans	θ_max_ = 27.0°, θ_min_ = 2.1°
Absorption correction: multi-scan (*SADABS*; Bruker, 2004)	*h* = −13→13
*T*_min_ = 0.969, *T*_max_ = 0.974	*k* = −11→11
25650 measured reflections	*l* = −24→24

### Refinement

**Table:**

Refinement on *F*^2^	Primary atom site location: structure-invariant direct methods
Least-squares matrix: full	Secondary atom site location: difference Fourier map
*R*[*F*^2^ > 2σ(*F*^2^)] = 0.045	Hydrogen site location: inferred from neighbouring sites
*wR*(*F*^2^) = 0.137	H atoms treated by a mixture of independent and constrained refinement
*S* = 1.09	*w* = 1/[σ^2^(*F*_o_^2^) + (0.0557*P*)^2^ + 0.7606*P*] where *P* = (*F*_o_^2^ + 2*F*_c_^2^)/3
3787 reflections	(Δ/σ)_max_ < 0.001
239 parameters	Δρ_max_ = 0.29 e Å^−^^3^
0 restraints	Δρ_min_ = −0.21 e Å^−^^3^

### Special details

**Table:**

Geometry. All esds (except the esd in the dihedral angle between two l.s. planes) are estimated using the full covariance matrix. The cell esds are taken into account individually in the estimation of esds in distances, angles and torsion angles; correlations between esds in cell parameters are only used when they are defined by crystal symmetry. An approximate (isotropic) treatment of cell esds is used for estimating esds involving l.s. planes.
Refinement. Refinement of F^2^ against ALL reflections. The weighted R-factor wR and goodness of fit S are based on F^2^, conventional R-factors R are based on F, with F set to zero for negative F^2^. The threshold expression of F^2^ > 2sigma(F^2^) is used only for calculating R-factors(gt) etc. and is not relevant to the choice of reflections for refinement. R-factors based on F^2^ are statistically about twice as large as those based on F, and R- factors based on ALL data will be even larger.

### Fractional atomic coordinates and isotropic or equivalent isotropic displacement parameters (Å^2^)

**Table:**

	*x*	*y*	*z*	*U*_iso_*/*U*_eq_	
O1	0.05279 (11)	0.22692 (16)	0.53826 (6)	0.0458 (3)	
O3	0.43975 (13)	0.34579 (16)	0.45820 (6)	0.0505 (4)	
O2	0.04279 (13)	0.03341 (17)	0.61440 (7)	0.0557 (4)	
N1	0.32532 (15)	−0.00285 (17)	0.57673 (7)	0.0427 (4)	
N2	0.43155 (15)	0.38257 (19)	0.57461 (8)	0.0454 (4)	
C9	0.25355 (16)	0.13927 (19)	0.58542 (8)	0.0342 (4)	
C7	0.15071 (16)	0.32733 (19)	0.51218 (8)	0.0368 (4)	
H7	0.1649	0.4112	0.5459	0.044*	
C10	0.27021 (15)	0.22222 (19)	0.51571 (8)	0.0334 (4)	
C13	0.29625 (17)	0.2330 (2)	0.64871 (8)	0.0381 (4)	
C19	0.38880 (16)	0.3193 (2)	0.51367 (8)	0.0378 (4)	
C18	0.38303 (17)	0.3521 (2)	0.64055 (9)	0.0404 (4)	
C11	0.27058 (18)	0.0894 (2)	0.46208 (9)	0.0401 (4)	
H11A	0.1898	0.0847	0.4353	0.048*	
H11B	0.3389	0.1028	0.4300	0.048*	
C14	0.2508 (2)	0.2060 (3)	0.71465 (9)	0.0522 (5)	
H14	0.1914	0.1280	0.7206	0.063*	
C6	0.11100 (17)	0.3972 (2)	0.44276 (9)	0.0400 (4)	
C1	0.17662 (19)	0.5259 (2)	0.42071 (9)	0.0436 (4)	
H1	0.2427	0.5654	0.4494	0.052*	
C8	0.10628 (17)	0.1232 (2)	0.58346 (9)	0.0403 (4)	
C12	0.2914 (2)	−0.0568 (2)	0.50569 (10)	0.0496 (5)	
H12A	0.2141	−0.1184	0.5052	0.059*	
H12B	0.3598	−0.1184	0.4876	0.059*	
C2	0.1465 (2)	0.5974 (2)	0.35712 (9)	0.0483 (5)	
C17	0.4237 (2)	0.4417 (2)	0.69755 (10)	0.0534 (5)	
H17	0.4813	0.5217	0.6918	0.064*	
C5	0.0112 (2)	0.3400 (2)	0.40112 (10)	0.0530 (5)	
H5	−0.0334	0.2535	0.4150	0.064*	
C3	0.0451 (2)	0.5388 (3)	0.31651 (10)	0.0568 (5)	
H3	0.0221	0.5854	0.2740	0.068*	
C16	0.3779 (2)	0.4109 (3)	0.76275 (10)	0.0624 (6)	
H16	0.4051	0.4704	0.8010	0.075*	
C20	0.2201 (3)	0.7356 (3)	0.33399 (12)	0.0682 (6)	
H20A	0.1865	0.7692	0.2890	0.102*	
H20B	0.3085	0.7086	0.3307	0.102*	
H20C	0.2123	0.8171	0.3674	0.102*	
C4	−0.0220 (2)	0.4130 (3)	0.33825 (11)	0.0627 (6)	
H4	−0.0905	0.3761	0.3105	0.075*	
C15	0.2922 (2)	0.2928 (3)	0.77163 (10)	0.0639 (6)	
H15	0.2624	0.2719	0.8158	0.077*	
C21	0.3205 (2)	−0.1220 (2)	0.63015 (11)	0.0571 (5)	
H21A	0.3721	−0.2079	0.6171	0.086*	
H21B	0.2338	−0.1553	0.6343	0.086*	
H21C	0.3524	−0.0814	0.6742	0.086*	
H2A	0.488 (2)	0.461 (3)	0.5713 (11)	0.058 (6)*	

### Atomic displacement parameters (Å^2^)

**Table:**

	*U*^11^	*U*^22^	*U*^33^	*U*^12^	*U*^13^	*U*^23^
O1	0.0362 (6)	0.0560 (8)	0.0455 (7)	−0.0010 (6)	0.0058 (5)	0.0056 (6)
O3	0.0578 (8)	0.0531 (8)	0.0422 (7)	−0.0136 (6)	0.0208 (6)	−0.0003 (6)
O2	0.0497 (8)	0.0598 (9)	0.0586 (8)	−0.0169 (7)	0.0140 (6)	0.0077 (7)
N1	0.0495 (9)	0.0392 (8)	0.0393 (8)	0.0024 (7)	0.0011 (6)	0.0039 (6)
N2	0.0459 (9)	0.0520 (9)	0.0384 (8)	−0.0206 (8)	0.0050 (6)	0.0006 (7)
C9	0.0368 (9)	0.0367 (8)	0.0294 (7)	−0.0049 (7)	0.0052 (6)	0.0018 (6)
C7	0.0386 (9)	0.0372 (9)	0.0350 (8)	−0.0003 (7)	0.0050 (7)	−0.0011 (7)
C10	0.0368 (8)	0.0365 (9)	0.0273 (7)	−0.0028 (7)	0.0051 (6)	−0.0004 (6)
C13	0.0397 (9)	0.0444 (9)	0.0306 (8)	−0.0034 (7)	0.0040 (7)	0.0009 (7)
C19	0.0403 (9)	0.0380 (9)	0.0356 (8)	−0.0039 (7)	0.0085 (7)	0.0025 (7)
C18	0.0421 (9)	0.0465 (10)	0.0326 (8)	−0.0052 (8)	0.0014 (7)	−0.0011 (7)
C11	0.0482 (10)	0.0398 (9)	0.0325 (8)	−0.0019 (8)	0.0048 (7)	−0.0033 (7)
C14	0.0586 (12)	0.0651 (13)	0.0335 (9)	−0.0121 (10)	0.0087 (8)	0.0021 (8)
C6	0.0464 (10)	0.0385 (9)	0.0349 (8)	0.0074 (8)	0.0008 (7)	−0.0044 (7)
C1	0.0537 (11)	0.0389 (9)	0.0378 (9)	0.0048 (8)	−0.0012 (8)	−0.0021 (7)
C8	0.0418 (9)	0.0436 (10)	0.0360 (8)	−0.0073 (8)	0.0067 (7)	−0.0022 (7)
C12	0.0635 (12)	0.0413 (10)	0.0439 (10)	0.0054 (9)	0.0022 (9)	−0.0039 (8)
C2	0.0645 (12)	0.0422 (10)	0.0385 (9)	0.0111 (9)	0.0053 (8)	−0.0008 (8)
C17	0.0569 (12)	0.0552 (12)	0.0475 (11)	−0.0128 (10)	−0.0047 (9)	−0.0069 (9)
C5	0.0566 (12)	0.0515 (11)	0.0500 (11)	−0.0028 (9)	−0.0066 (9)	−0.0009 (9)
C3	0.0751 (14)	0.0579 (12)	0.0368 (10)	0.0158 (11)	−0.0030 (9)	0.0020 (9)
C16	0.0726 (15)	0.0767 (15)	0.0374 (10)	−0.0030 (12)	−0.0059 (9)	−0.0158 (10)
C20	0.0925 (18)	0.0582 (13)	0.0540 (12)	0.0012 (12)	0.0053 (12)	0.0138 (10)
C4	0.0667 (14)	0.0691 (15)	0.0507 (12)	0.0009 (12)	−0.0169 (10)	−0.0063 (11)
C15	0.0738 (15)	0.0882 (17)	0.0303 (9)	−0.0080 (13)	0.0075 (9)	−0.0051 (10)
C21	0.0636 (13)	0.0513 (12)	0.0559 (12)	0.0000 (10)	−0.0032 (10)	0.0159 (9)

### Geometric parameters (Å, º)

**Table:**

O1—C8	1.353 (2)	C14—H14	0.9300
O1—C7	1.451 (2)	C6—C5	1.378 (3)
O3—C19	1.2292 (19)	C6—C1	1.387 (3)
O2—C8	1.197 (2)	C1—C2	1.389 (2)
N1—C21	1.456 (2)	C1—H1	0.9300
N1—C9	1.458 (2)	C12—H12A	0.9700
N1—C12	1.465 (2)	C12—H12B	0.9700
N2—C19	1.347 (2)	C2—C3	1.385 (3)
N2—C18	1.404 (2)	C2—C20	1.503 (3)
N2—H2A	0.91 (2)	C17—C16	1.381 (3)
C9—C13	1.510 (2)	C17—H17	0.9300
C9—C10	1.531 (2)	C5—C4	1.389 (3)
C9—C8	1.548 (2)	C5—H5	0.9300
C7—C6	1.501 (2)	C3—C4	1.374 (3)
C7—C10	1.548 (2)	C3—H3	0.9300
C7—H7	0.9800	C16—C15	1.378 (3)
C10—C19	1.504 (2)	C16—H16	0.9300
C10—C11	1.543 (2)	C20—H20A	0.9600
C13—C14	1.386 (2)	C20—H20B	0.9600
C13—C18	1.390 (2)	C20—H20C	0.9600
C18—C17	1.390 (2)	C4—H4	0.9300
C11—C12	1.529 (3)	C15—H15	0.9300
C11—H11A	0.9700	C21—H21A	0.9600
C11—H11B	0.9700	C21—H21B	0.9600
C14—C15	1.379 (3)	C21—H21C	0.9600
			
C8—O1—C7	109.89 (13)	C6—C1—C2	121.97 (18)
C21—N1—C9	119.19 (15)	C6—C1—H1	119.0
C21—N1—C12	114.09 (16)	C2—C1—H1	119.0
C9—N1—C12	105.69 (13)	O2—C8—O1	121.69 (17)
C19—N2—C18	125.42 (15)	O2—C8—C9	128.41 (17)
C19—N2—H2A	116.1 (14)	O1—C8—C9	109.88 (14)
C18—N2—H2A	117.9 (14)	N1—C12—C11	105.24 (14)
N1—C9—C13	114.29 (14)	N1—C12—H12A	110.7
N1—C9—C10	102.71 (12)	C11—C12—H12A	110.7
C13—C9—C10	113.74 (13)	N1—C12—H12B	110.7
N1—C9—C8	116.04 (14)	C11—C12—H12B	110.7
C13—C9—C8	109.18 (13)	H12A—C12—H12B	108.8
C10—C9—C8	99.93 (13)	C3—C2—C1	117.75 (19)
O1—C7—C6	111.93 (14)	C3—C2—C20	121.27 (18)
O1—C7—C10	102.31 (13)	C1—C2—C20	120.97 (19)
C6—C7—C10	117.98 (13)	C16—C17—C18	119.49 (19)
O1—C7—H7	108.1	C16—C17—H17	120.3
C6—C7—H7	108.1	C18—C17—H17	120.3
C10—C7—H7	108.1	C6—C5—C4	119.4 (2)
C19—C10—C9	114.33 (13)	C6—C5—H5	120.3
C19—C10—C11	112.09 (13)	C4—C5—H5	120.3
C9—C10—C11	103.29 (13)	C4—C3—C2	120.85 (19)
C19—C10—C7	109.63 (14)	C4—C3—H3	119.6
C9—C10—C7	101.11 (12)	C2—C3—H3	119.6
C11—C10—C7	115.91 (14)	C15—C16—C17	120.59 (19)
C14—C13—C18	118.69 (16)	C15—C16—H16	119.7
C14—C13—C9	122.39 (16)	C17—C16—H16	119.7
C18—C13—C9	118.92 (14)	C2—C20—H20A	109.5
O3—C19—N2	121.95 (16)	C2—C20—H20B	109.5
O3—C19—C10	121.17 (15)	H20A—C20—H20B	109.5
N2—C19—C10	116.77 (14)	C2—C20—H20C	109.5
C13—C18—C17	120.52 (16)	H20A—C20—H20C	109.5
C13—C18—N2	120.33 (15)	H20B—C20—H20C	109.5
C17—C18—N2	119.15 (17)	C3—C4—C5	120.8 (2)
C12—C11—C10	105.24 (13)	C3—C4—H4	119.6
C12—C11—H11A	110.7	C5—C4—H4	119.6
C10—C11—H11A	110.7	C16—C15—C14	119.58 (19)
C12—C11—H11B	110.7	C16—C15—H15	120.2
C10—C11—H11B	110.7	C14—C15—H15	120.2
H11A—C11—H11B	108.8	N1—C21—H21A	109.5
C15—C14—C13	121.12 (19)	N1—C21—H21B	109.5
C15—C14—H14	119.4	H21A—C21—H21B	109.5
C13—C14—H14	119.4	N1—C21—H21C	109.5
C5—C6—C1	119.21 (17)	H21A—C21—H21C	109.5
C5—C6—C7	122.61 (17)	H21B—C21—H21C	109.5
C1—C6—C7	118.17 (16)		
			
C21—N1—C9—C13	63.0 (2)	C14—C13—C18—N2	179.77 (18)
C12—N1—C9—C13	−167.01 (14)	C9—C13—C18—N2	−1.1 (3)
C21—N1—C9—C10	−173.31 (15)	C19—N2—C18—C13	9.7 (3)
C12—N1—C9—C10	−43.33 (17)	C19—N2—C18—C17	−170.84 (18)
C21—N1—C9—C8	−65.4 (2)	C19—C10—C11—C12	108.42 (17)
C12—N1—C9—C8	64.55 (18)	C9—C10—C11—C12	−15.13 (18)
C8—O1—C7—C6	156.13 (14)	C7—C10—C11—C12	−124.70 (16)
C8—O1—C7—C10	28.85 (16)	C18—C13—C14—C15	−1.3 (3)
N1—C9—C10—C19	−86.79 (16)	C9—C13—C14—C15	179.6 (2)
C13—C9—C10—C19	37.3 (2)	O1—C7—C6—C5	−16.5 (2)
C8—C9—C10—C19	153.46 (14)	C10—C7—C6—C5	101.8 (2)
N1—C9—C10—C11	35.28 (16)	O1—C7—C6—C1	162.34 (14)
C13—C9—C10—C11	159.32 (14)	C10—C7—C6—C1	−79.3 (2)
C8—C9—C10—C11	−84.48 (15)	C5—C6—C1—C2	−1.3 (3)
N1—C9—C10—C7	155.54 (13)	C7—C6—C1—C2	179.75 (16)
C13—C9—C10—C7	−80.42 (16)	C7—O1—C8—O2	176.05 (16)
C8—C9—C10—C7	35.78 (15)	C7—O1—C8—C9	−5.39 (18)
O1—C7—C10—C19	−161.30 (12)	N1—C9—C8—O2	48.4 (2)
C6—C7—C10—C19	75.41 (18)	C13—C9—C8—O2	−82.4 (2)
O1—C7—C10—C9	−40.25 (15)	C10—C9—C8—O2	157.98 (18)
C6—C7—C10—C9	−163.54 (14)	N1—C9—C8—O1	−129.98 (15)
O1—C7—C10—C11	70.59 (16)	C13—C9—C8—O1	99.13 (16)
C6—C7—C10—C11	−52.7 (2)	C10—C9—C8—O1	−20.45 (17)
N1—C9—C13—C14	−85.5 (2)	C21—N1—C12—C11	166.51 (16)
C10—C9—C13—C14	156.99 (17)	C9—N1—C12—C11	33.62 (19)
C8—C9—C13—C14	46.3 (2)	C10—C11—C12—N1	−10.3 (2)
N1—C9—C13—C18	95.38 (19)	C6—C1—C2—C3	1.9 (3)
C10—C9—C13—C18	−22.2 (2)	C6—C1—C2—C20	−179.25 (18)
C8—C9—C13—C18	−132.79 (17)	C13—C18—C17—C16	0.4 (3)
C18—N2—C19—O3	−176.51 (18)	N2—C18—C17—C16	−179.0 (2)
C18—N2—C19—C10	7.3 (3)	C1—C6—C5—C4	−0.4 (3)
C9—C10—C19—O3	153.12 (16)	C7—C6—C5—C4	178.49 (19)
C11—C10—C19—O3	36.0 (2)	C1—C2—C3—C4	−0.9 (3)
C7—C10—C19—O3	−94.19 (19)	C20—C2—C3—C4	−179.7 (2)
C9—C10—C19—N2	−30.6 (2)	C18—C17—C16—C15	−0.2 (3)
C11—C10—C19—N2	−147.74 (16)	C2—C3—C4—C5	−0.8 (3)
C7—C10—C19—N2	82.07 (18)	C6—C5—C4—C3	1.4 (3)
C14—C13—C18—C17	0.3 (3)	C17—C16—C15—C14	−0.8 (4)
C9—C13—C18—C17	179.47 (17)	C13—C14—C15—C16	1.5 (4)

### Hydrogen-bond geometry (Å, º)

**Table:**

*D*—H···*A*	*D*—H	H···*A*	*D*···*A*	*D*—H···*A*
N2—H2*A*···O3^i^	0.91 (2)	1.93 (2)	2.802 (2)	159.6 (19)
C5—H5···O2^ii^	0.93	2.55	3.303 (3)	138

Symmetry codes: (i) −*x*+1, −*y*+1, −*z*+1; (ii) −*x*, −*y*, −*z*+1.
