# Supplementary figures and images for: Crystal structure of 14-methyl-11-(3-methyl­phen­yl)-12-oxa-8,14-di­aza­tetra­cyclo­[8.3.3.01,10.02,7]hexa­deca-2(7),3,5-triene-9,13-dione
Source: Acta Crystallogr E Crystallogr Commun. 2015 May 7;71(Pt 6):o379–80. doi: 10.1107/S2056989015008129 (PMC4459358; doi:10.1107/S2056989015008129)

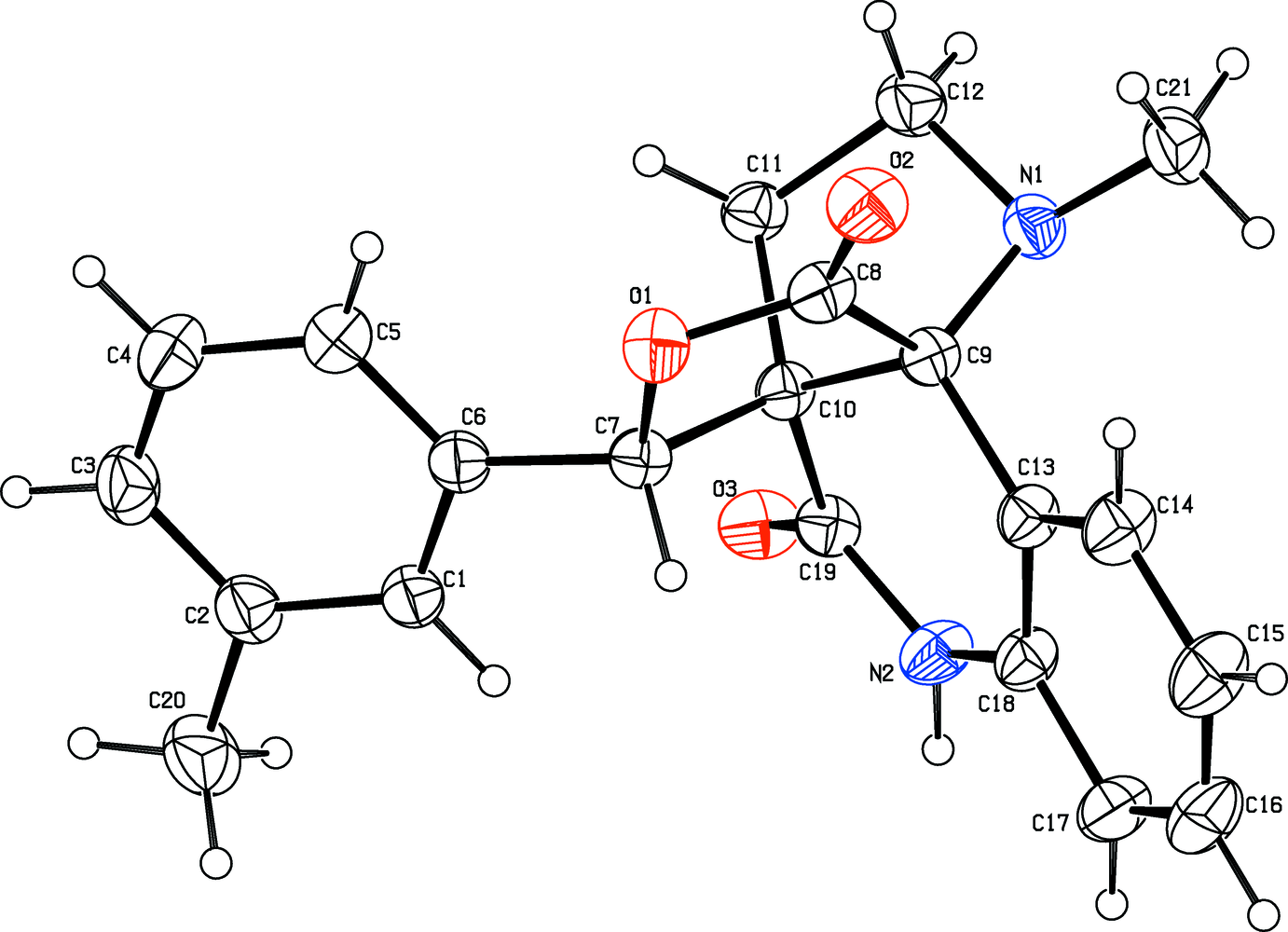

Supplement: Supplementary file 4 [file e-71-0o379-fig1.tif]

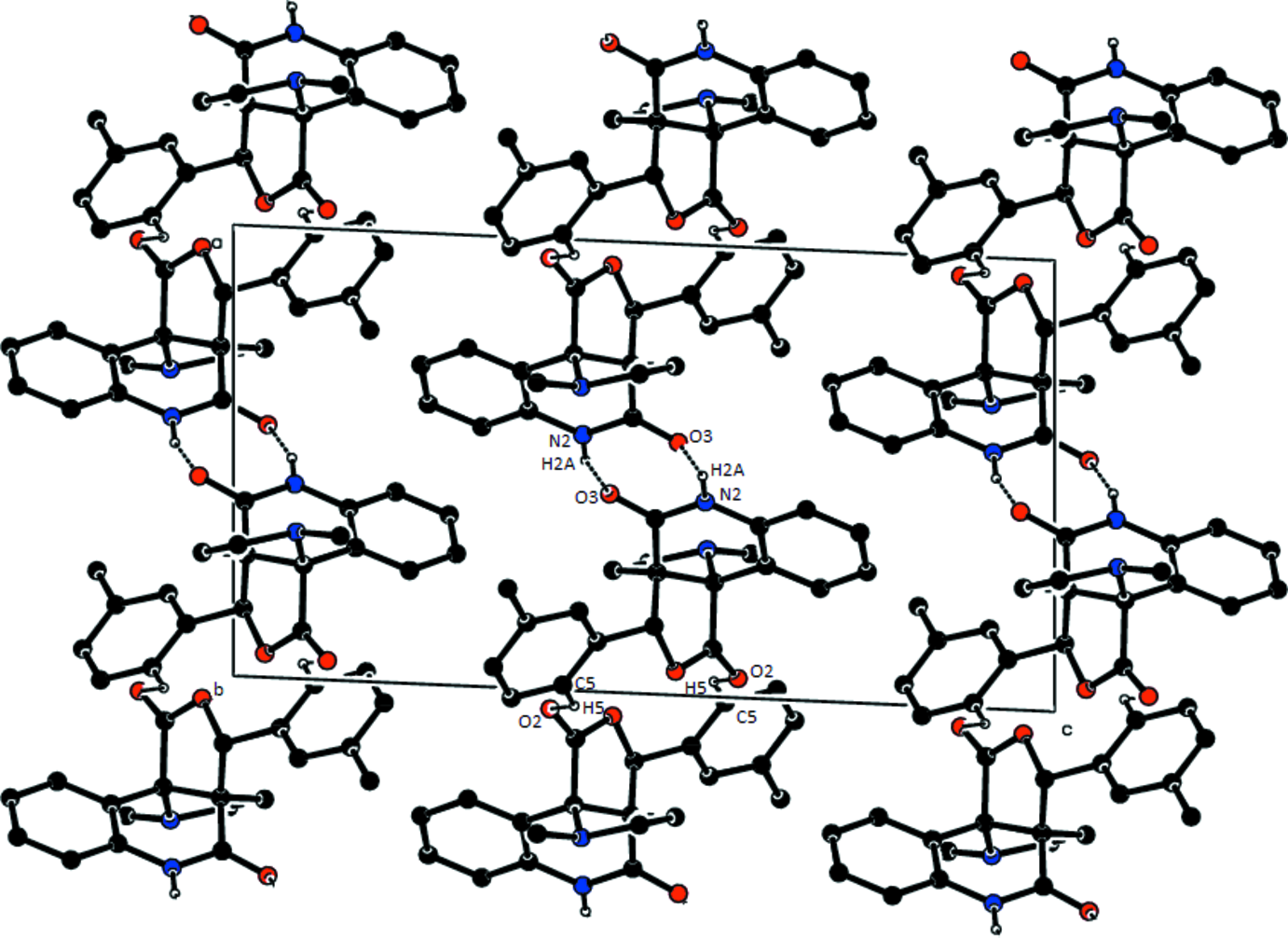

Supplement: Supplementary file 5 [file e-71-0o379-fig2.tif]
